# The application of acupuncture in cardiopathy: A bibliometric analysis based on Web of Science across ten recent years

**DOI:** 10.3389/fcvm.2022.920491

**Published:** 2022-09-06

**Authors:** Xiao Li, Zihan Yin, Fayang Ling, Qianhua Zheng, Xiang Li, Wenchuan Qi, Fanrong Liang

**Affiliations:** ^1^School of Acu-Mox and Tuina/The Third Teaching Hospital, Chengdu University of Traditional Chinese Medicine, Chengdu, Sichuan, China; ^2^Acupuncture and Tuina School/The Third Teaching Hospital, Chengdu University of Traditional Chinese Medicine/Clinical Research Center for Acupuncture and Moxibustion in Sichuan Province, Chengdu, Sichuan, China

**Keywords:** acupuncture, cardiopathy, cardiovascular disease, bibliometric analysis, CiteSpace

## Abstract

**Background:**

With high morbidity and mortality, cardiopathy is a major component of cardiovascular disease, causing a huge burden of disease to public health worldwide. In recent years, research on acupuncture treatment of cardiopathies has been increasing. However, no bibliometric analysis has been conducted to systematically describe the research progress and hotspots in this field. Therefore, this study aimed to conduct a bibliometric analysis of the relevant literature to explore the current status and future development of acupuncture for cardiopathies.

**Methods:**

The Web of Science (WoS) Core Collection Database was searched for literature related to acupuncture therapies for cardiopathies from 2011 to 2021. Using CiteSpace 5.8 R3, cooperation network diagrams of authors, institutions, countries and journals, keyword co-occurrences, and clustering were performed and analyzed.

**Results:**

A total of 321 studies were included. Overall, the number of annual publications increased yearly. These publications came from 31 countries or regions, of which China and the United States made the greatest contributions. In total, 333 authors from 258 institutions participated in this field, and Beijing University of Chinese Medicine and Professor Fanrong Liang were the most published institution and author, respectively. *Evidence-based Complementary and Alternative Medicine* published the largest number of articles, and *CIRCULATION* was the most commonly cited journal. Based on co-occurrences and cluster analysis of 257 keywords, three research frontiers and hotspots were identified: acupuncture for blood pressure regulation, acupuncture for coronary heart disease, and acupuncture for regulation of heart rate. In these three research frontiers, the rostral ventrolateral medulla (RVLM) and autonomic nervous system (ANS) are the most popular mechanisms.

**Conclusion:**

A stable development trend has formed in this field. Further research should focus on the role of acupuncture therapies in the treatment of hypertension or hypertensive heart disease, coronary heart disease, and arrhythmia based on the mechanisms related to the RVLM and ANS.

## Introduction

Cardiovascular disease is the leading contributor to the burden of disease worldwide. With population growth and aging, the number of cardiovascular diseases worldwide has almost doubled from 271 million in 1990 to 523 million in 2019 (1). As a major branch of cardiovascular disease, heart disease includes congenital heart disease, hypertensive heart disease, pulmonary heart disease, coronary heart disease, arrhythmia, cardiomyopathy, pericardial disease, and heart valve disease. Most of these diseases are characterized by acute onset, such as coronary heart disease, which often manifests as sudden cardiac arrest or even sudden cardiac death (2). Sudden cardiac death (SCD) has led to approximately 230∼350 thousand deaths per year in the United States over the past 20∼30 years (3). During 2016 in China, the age-standardized prevalence rate of ischemic heart disease was 15.07‰, while that of atrial fibrillation and flutter was 6.05‰, and that of hypertensive heart disease was 3.22‰ (4). There were 40.7 cardiac deaths in China per 100,000 person-years from 1980 to 2016 (5). These gloomy statistics represent a huge challenge for the world.

As a treasure in traditional Chinese medicine, acupuncture has become one of the most widely accepted alternative medicine methods worldwide. Disease spectrum analysis of 2,471 systematic evaluations of acupuncture therapy from 2000 to 2020 in the Web of Science (WoS) database showed that acupuncture therapy covers 77 kinds of illnesses in 12 fields (6), indicating that acupuncture therapy has broad spectrum characteristics. In recent years, the mechanism of acupuncture in analgesia, anti-inflammation, and nerve regulation has been the subject of abundant mechanistic research (7–9), which provides a theoretical basis for research related to acupuncture therapies and cardiopathies.

CiteSpace is a software for visual analysis of literature to understand the past and present development of relevant fields and to identify the most active research frontiers and development trends. CiteSpace, which stands for “citation space,” is a citation visualization software that focuses on the investigation of potential knowledge contained in scientific analysis and was gradually developed against the background of scientometrics and data visualization. Because the structure, law, and distribution of scientific knowledge are presented through visualization, the visualization graph obtained through the examination of such methods is also called the “scientific knowledge map.” Cooperative network analysis, co-occurrence analysis, and co-citation analysis of the original literature can be performed based on the Java development environment. Recently, bibliometric analyses of acupuncture and obstetrical and gynecological diseases (10), migraine (11), insomnia (12), knee osteoarthritis (13), and cancer (14) have been reported, revealing the research progress and frontiers of acupuncture and related diseases. However, no report concerning the application of acupuncture in the treatment of cardiopathies based on bibliometric analysis has been found.

Therefore, in the present study, we aimed to explore the frontiers and development trends of acupuncture for cardiopathies based on co-occurrence network diagrams of authors, academic institutions, countries and scientific journals, keyword co-occurrences, and clustering over the past 10 years via CiteSpace.

## Methods

### Data collection

We extracted literature from the SCI-EXPANDED database in the WoS Core Collection via the Chengdu University of Traditional Chinese Medicine Library’s website. The search strategy (Table 1) included the topics “acupuncture therapy” and “cardiopathy” from January 1, 2011 to December 31, 2021, and we downloaded all data within 1 day on February 15, 2022. There was no restriction on language, and we retrieved 801 studies. After eliminating meeting abstracts (14), editorial material (18), book chapters (3), letters (11), corrections (1) and articles not related to the search topic (433), we included 321 records (see the Supplementary Material), including 255 articles and 66 reviews, for further visualization and analysis. We saved these papers in a text document and named it “download_txt.”

**TABLE 1:** Search strategy in this study.

Set	Result	Search query
#1	17,426	TS = (“Acupuncture” OR “Pharmacoacupuncture Treatment” OR “Acupotomy” OR “Electroacupuncture” OR “Electro-acupuncture” OR “Body Acupuncture” OR “Manual Acupuncture” OR “Auricular” OR “Auricular Acupuncture” OR “Auricular Needle” OR “Acupuncture Point” OR “Ear Acupuncture” OR “Warm Acupuncture” OR “Moxibustion” OR “Moxabustion” OR “Acupoint Injection” OR “Catgut Embedding” OR “Catgut Implantation at Acupoint” OR “Embedding Thread”)
#2	735,221	TS = (“Cardiovascular Disease” OR “Heart Disease” OR “Coronary Heart Disease” OR “Coronary Artery Disease” OR “Angina” OR “Pectoris” OR “Angina Pectoris” OR “Myocardial Ischemia” OR “Myocardium Ischemia” OR “Heart Attack” OR “Cardiopathy” OR “Cardiomyopathy” OR “Hypertension” OR “High Blood Pressure” OR “Hypertensive heart disease” OR “Myocardial Infraction” OR “MI” OR “STEMI” OR “Arrhythmia” OR “Irrhythmia” OR “Arhythmia” OR “Irregular Pulse” OR “Carditis” OR “Myocarditis” OR “Dilated Cardiomyopathy” OR “Hypertrophic Cardiomyopathy” OR “Hypertrophelloc Cardiomyopathy”)
#3	22,805,753	PY = (2001–2021)
#4	801	#3 AND #2 AND #1

### Data analysis

We employed CiteSpace 5.8.R3, which contains a bibliometric technique to describe the characteristics of the included literature. First, we constructed cooperation network diagrams of authors, academic institutions, countries and scientific journals to describe individual contributions and cooperation among these entities. In addition, we performed keyword co-occurrence analysis, keyword clustering analysis, and citation burst analysis to identify research directions and hotspots. The software parameters were as follows: (1) time slicing: from January 2011 to December 2021, the number of years per slice was 1; (2) each node type was selected at a time; (3) selection criteria: g-index: *k* = 25; and (4) pruning: pathfinder or minimum spanning tree. The g-index measures the research achievements of an independent individual or unit. In CiteSpace, on the basis of increasing the scale Factor k, we used a modified g-index algorithm to rank and extract a specific number of independent individuals or units. The “pruning” algorithm is employed to simplify lines in a network to reduce the network’s density and improve its readability. “Pathfinder” and “minimum spanning tree” are the most commonly used network clipping methods in CiteSpace (15).

The visualization maps consisted of tree rings and lines connecting the tree rings. Each tree ring (node) represents a unit (e.g., an author, a country or region, a journal), and the links represent the cooperation or co-occurrence between them. A larger tree ring denotes more publications or a larger frequency of occurrence of the unit, and thicker lines indicate more cooperation or co-occurrence between them. The color of the tree rings and lines provides a perspective of evolution over time, in which a shift in tone from cool to warm signals the time from far to near. Meanwhile, CiteSpace uses “betweenness centrality” to describe the umbilical role of a node and marks it with a purple ring.

## Results

### Annual publication outputs and time trend

**FIGURE 1:**
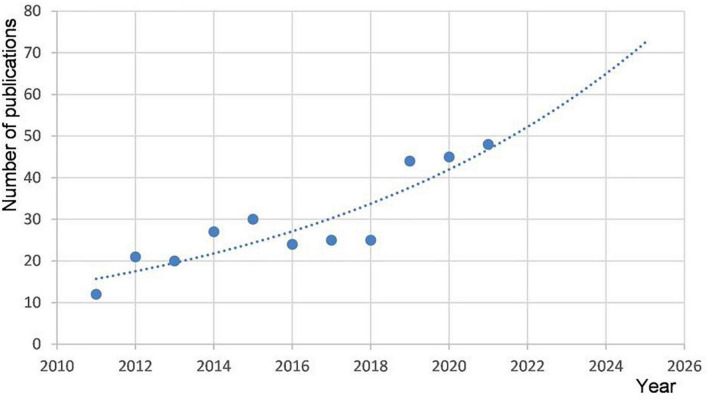
Annual publications from 2011 to 2021 and the time trend of acupuncture on cardiopathy.

Since 12 articles emerged in 2011, the number of studies published on this topic rose with fluctuations. From 2016 to 2018, the number of articles declined, but by a small margin. In 2019, the annual publication output rose significantly, and in 2021, the output increased continuously (Figure 1). The time curve suggests a trend of steady growth in the number of publications in the near future.

### Distribution of countries/regions

**FIGURE 2:**
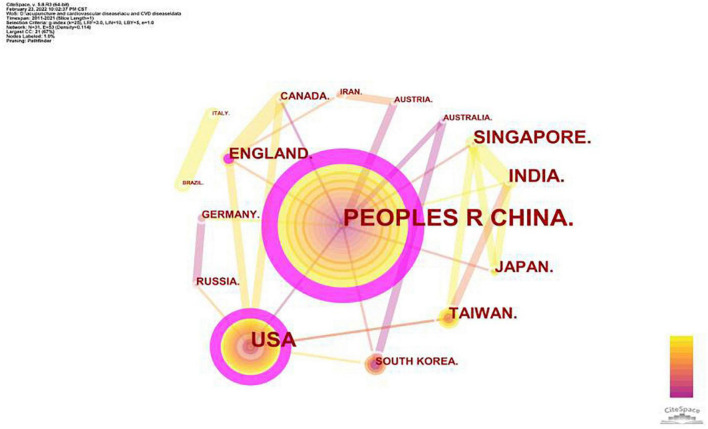
Network map of co-authorship between countries or regions about acupuncture on cardiopathy.

We used CiteSpace to visualize the numbers of articles published in each country or region about acupuncture therapy for the treatment of cardiopathies. The map of country/region distribution consisted of 31 nodes and 56 links, indicating that 31 countries or regions have made contributions to the research on acupuncture therapy for cardiopathies (Figure 2). The People’s Republic of China (PRC) was number 1 with an absolute advantage (204 publications), followed by the United States (47 publications), Taiwan (26 publications), South Korea (25 publications), and Japan (19 publications). The top two countries in terms of centrality were China (0.47) and the United States (0.25), and other countries or regions were less than 0.10 (Table 2). This finding suggests that China and the United States play crucial roles in research in this field, and they have both formed their own networks of international cooperation. However, the thin line between China and the United States implies that the academic exchanges between the two countries are not rich enough.

**TABLE 2:** The top 10 active countries/regions.

Rank	Country/region	Publications	Centrality
1	China	204	0.47
2	United States	47	0.25
3	Taiwan	26	0.01
4	South Korea	25	0.06
5	Japan	19	0.00
6	Austria	6	0.00
7	Brazil	5	0.00
8	Australia	5	0.00
9	Turkey	5	0.00
10	England	4	0.06

### Distribution of institutions

**FIGURE 3:**
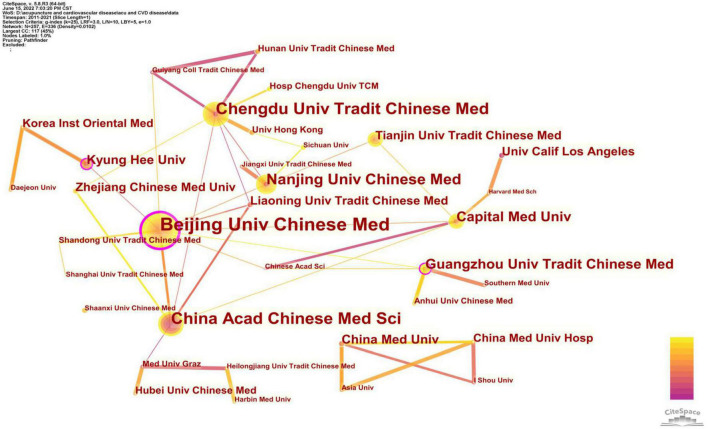
Network map of co-authorship between institutions about acupuncture on cardiopathy.

We performed the co-occurrence analysis of institutions using CiteSpace, resulting in 258 nodes and 333 links, which means that 258 institutions have participated in research on acupuncture and cardiopathy (Figure 3). The top ten institutions were Beijing University of Chinese Medicine (39), Chengdu University of Traditional Chinese Medicine (28), China Academy of Chinese Medical Sciences (26), Nanjing University of Chinese Medicine (20), Guangzhou University of Chinese Medicine (17), Capital Medical University (16), University of California System (14), China Medical University (14), Kyung Hee University (13), and Tianjin University of Traditional Chinese Medicine (13). For centrality, the top three institutions were Beijing University of Chinese Medicine (0.15), Kyung Hee University (0.11), and Guangzhou University of Chinese Medicine (0.11), while the others were no more than 0.10 (Table 3). The high centrality nodes are marked by purple circles in the mapping. Eight of the top institutions with the highest publication numbers came from China, which is consistent with the analysis of national contributions.

**TABLE 3:** The top 10 active institutions.

Rank	Institution	Publications	Centrality
1	Beijing University of Chinese Medicine	39	0.15
2	Chengdu University of Traditional Chinese Medicine	28	0.06
3	China Academy of Chinese Medical Sciences	26	0.09
4	Nanjing University of Chinese Medicine	20	0.09
5	Guangzhou University of Chinese Medicine	17	0.11
6	Capital Medical University	16	0.10
7	University of California System	14	0.03
8	China Medical University	14	0.01
9	Kyung Hee University	13	0.11
10	Tianjin University of Traditional Chinese Medicine	13	0.05

University of California System (University of California System) includes University of California, Los Angeles (10 publications), University of California, Irvine and other campuses.

### Authors and co-cited authors

**FIGURE 4:**
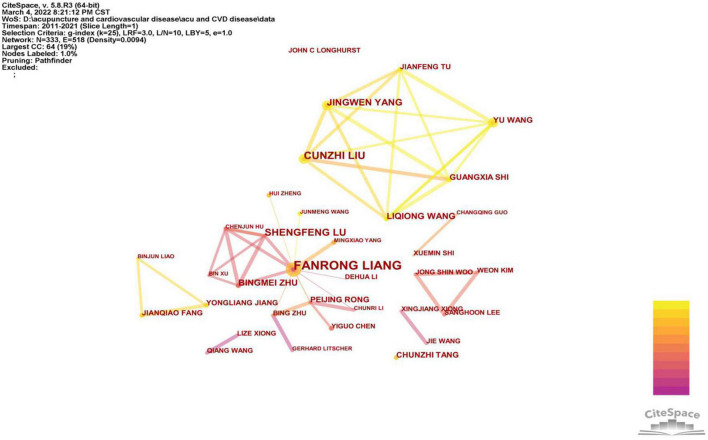
Network map of co-authorship between authors about acupuncture on cardiopathy.

A total of 333 authors have taken part in research on acupuncture in relation to cardiopathies (Figure 4). Fanrong Liang from Chengdu University of Traditional Chinese Medicine ranked first with 21 publications, followed by Cunzhi Liu (12), Shengfeng Lu (12), Yu Wang (12), Jingwen Yang (11), Guangxia Shi (9), Liqiong Wang (7), Bingmei Zhu (7), Peijing Rong (7), and Chunzhi Tang (6). A noteworthy feature of this outcome is that the centrality of the authors was relatively low (less than 0.10), indicating that these authors have not established a close and influential cooperative network (Table 4A). Five of the top ten authors come from Beijing University of Chinese Medicine, and all top ten authors come from China. These results echo the analysis of the contributions of countries and institutions.

**TABLE 4a:** The top 10 active authors.

Rank	Author	Institution	Country	Count	Centrality
1	Fanrong Liang	Chengdu University of Traditional Chinese Medicine	China	21	0.05
2	Cunzhi Liu	Beijing University of Chinese Medicine	China	12	0.00
3	Shengfeng Lu	Nanjing University of Chinese Medicine	China	12	0.02
4	Yu Wang	Beijing University of Chinese Medicine	China	12	0.00
5	Jingwen Yang	Beijing University of Chinese Medicine	China	11	0.00
6	Guangxia Shi	Beijing University of Chinese Medicine	China	9	0.00
7	Liqiong Wang	Beijing University of Chinese Medicine	China	7	0.00
8	Bingmei Zhu	Sichuan University	China	7	0.01
9	Peijing Rong	China Academy of Chinese Medical Science	China	7	0.00
10	Chunzhi Tang	Guangzhou University of Chinese Medicine	China	6	0.00

**FIGURE 5:**
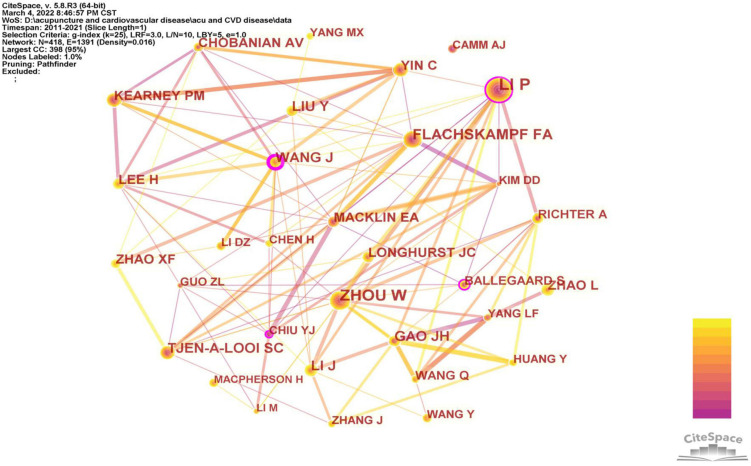
Network map of co-cited authors about acupuncture on cardiopathy.

Among the 418 co-cited authors (Figure 5), three were cited more than 50 times. The top ten co-cited authors are shown in Table 4B. Li Peng from the University of California (Irvine) ranked first, with 74 citations, followed by Zhou Wei (62), Flachskampf FA (50), Tjen-A-Looi SC (36), Gao Junhong (35), Wang Jie (34), Li Jing (34), Zhao Ling (33), Kearney PM (32), and Lee H (32). Nine of the top ten co-cited authors came from the United States and China, indicating that publications in the United States and China provide important bases for further research on acupuncture treatment of cardiopathies.

**TABLE 4b:** The top 10 co-cited authors.

Rank	Co-cited author	Institution	Country	Count	Centrality
1	Li Peng (Li P)	University of California System (Irvine)	United States	74	0.13
2	Zhou Wei (Zhou W)	University of California System (Los Angeles)	United States	62	0.09
3	Flachskampf FA	Nanjing University of Chinese Medicine	China	50	0.07
4	Tjen-A-Looi SC	University of California System (Irvine)	United States	36	0.06
5	Gao Junhong (Gao JH)	China Academy of Chinese Medical Science	China	35	0.09
6	Wang Jie (Wang J)	China Academy of Chinese Medical Science	China	34	0.20
7	Li Jing (Li J)	Guangzhou University of Chinese Medicine	China	34	0.05
8	Zhao Ling (Zhao L)	Chengdu University of Traditional Chinese Medicine	China	33	0.03
9	Kearney PM	Tulane University	United States	32	0.09
10	Lee H	Kyung Hee University	South Korea	32	0.07

### Journals and co-cited journals

All 321 articles were published in 104 journals. *Evidence-based Complementary and Alternative Medicine (EVID-BASED COMPL ALT)* was the most productive journal, with 50 publications, more than twice that of the runner-up. The top ten most popular journals are presented in Table 5. The IF score of these journals was no more than 4, and the highest score was *BMC Complementary and Alternative Medicine*, with an IF score of 3.659.

**TABLE 5:** The top 10 popular journals and cited journals.

Rank	Journal	Count	IF (2021)	Co-cited journal	Count	IF (2021)
1	EVID-BASED COMPL ALT	50	2.629	CIRCULATION	199	29.690
2	Medicine	21	1.889	EVID-BASED COMPL ALT	185	2.629
3	ACUPUNCT MED	20	2.267	ZHONG GUO ZHEN JIU	119	–
4	J TRADIT CHIN MED	17	0.848	PLOS ONE	103	3.24
5	ACUPUNCTURE ELECTRO	11	0.143	AM J PHYSIOL-HEART C	101	4.733
6	BMC COMPLEM ALTERN M	10	3.659	EUR HEART J	95	4.696
7	CHIN J INTEGR MED	10	1.978	LANCET	95	79.321
8	TRIALS	9	2.279	INT J CARDIOL	95	4.164
9	COMPLEMENT THER MED	8	2.446	ZHEN CI YAN JIU	94	–
10	J ALTERN COMPLEM MED	8	2.579	AM J CHINESE MED	93	4.667

**FIGURE 6:**
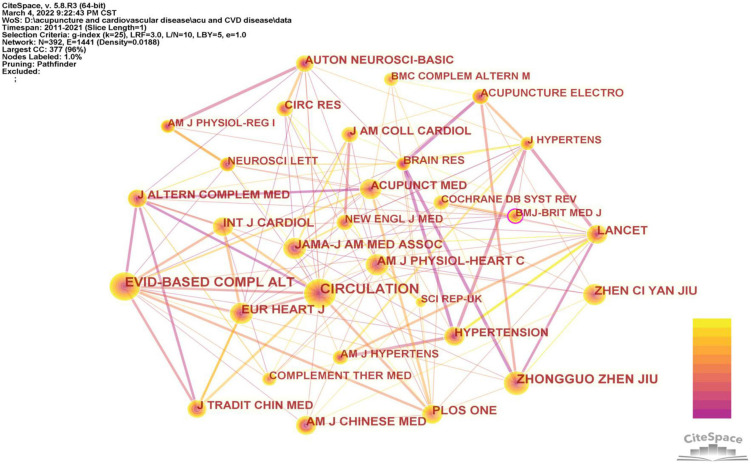
Network map of co-cited journals about acupuncture on cardiopathy.

The top ten co-cited academic journals with more than 90 citations are displayed in Table 5. *Circulation* ranked first with 199 citations, followed by *EVID-BASED COMPL ALT* (185 citations), *Zhongguo zhen jiu* (119 citations), *PLOS one* (103 citations), and the *American Journal of Physiology-Heart and Circulatory Physiology* (101 citations). Figure 6 outlines the network map of co-cited journals with more than 50 outputs.

### Keyword co-occurrences and clusters

**FIGURE 7:**
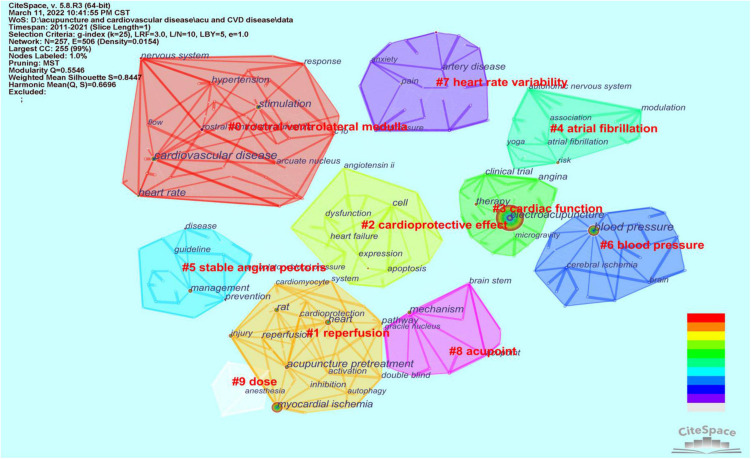
257 keywords were classified into 10 clusters.

**FIGURE 8:**
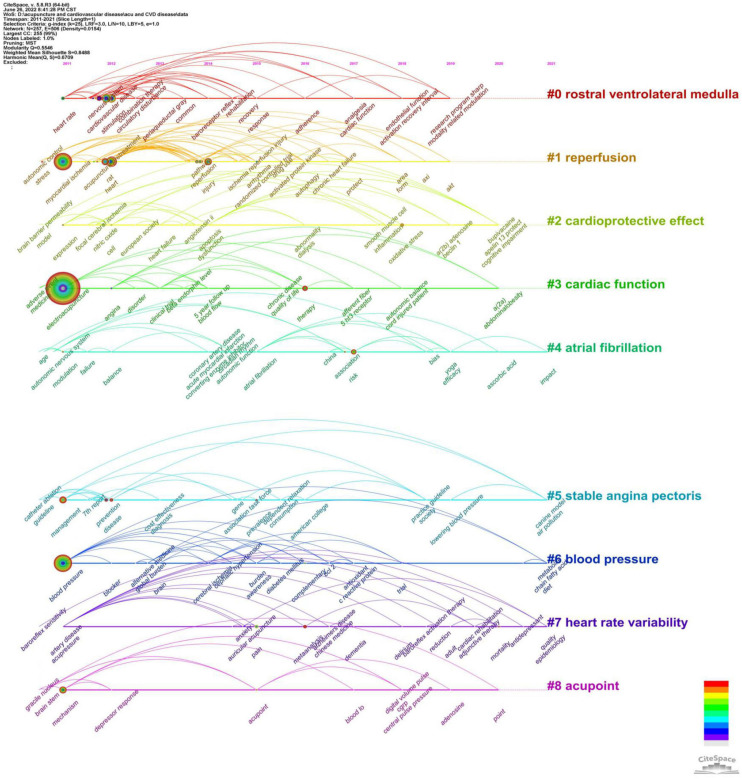
Timeline view of the keywords about acupuncture on cardiopathy. The top nine clusters were arranged on a horizontal timeline, and the direction of time points to the right from 2011 to 2021. The horizontal lines are timelines, with different color in each cluster. The tree rings represent occurrence of keywords, and the larger rings represent more frequency of occurrence. The camber line above the horizontal line represents the co-occurrence relationship between keywords.

**FIGURE 9:**
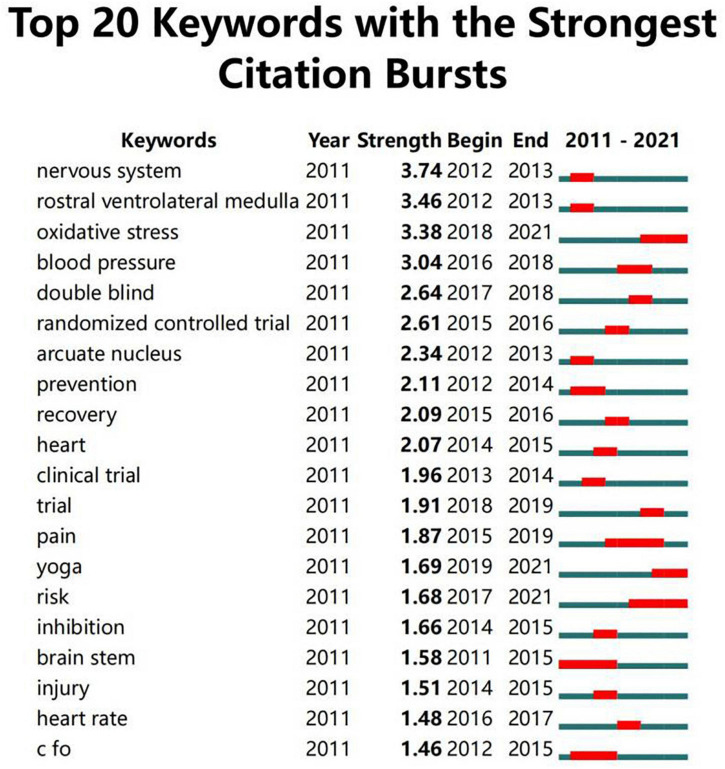
The top 20 keywords with the strongest citation bursts about acupuncture on cardiopathy.

Keywords summarize the research area of an article. The analysis of keywords helps to identify current research hotspots. Table 6 depicts the top 20 high-frequency keywords. More specific and representative keywords include blood pressure, myocardial ischemia, nervous system, rostral ventrolateral medulla, heart rate, reperfusion and angina. “Electroacupuncture,” “blood pressure,” and “myocardial ischemia” ranked as the top three with frequencies larger than 40. A total of 257 keywords in the 321 publications were classified into 10 clusters: “rostral ventrolateral medulla,” “reperfusion,” “cardioprotective effect,” “cardiac function,” “atrial fibrillation,” “stable angina pectoris,” “blood pressure,” “heart rate variability,” “acupoint,” and “dose,” as shown in Figure 7 with different colors. We used a timeline view in Figure 8 to demonstrate the evolution of keywords over time. The top 9 clusters were arranged on a horizontal timeline, and the direction of time points to the right. In each cluster, the revolution of relevant keywords is clearly presented over time from 2011 to 2021. The top 20 keywords with the strongest citation bursts in this field from 2011 to 2021 are portrayed in Figure 9. “Nervous system,” “rostral ventrolateral medulla,” “oxidative stress,” and “blood pressure” were listed as the top four with the strongest citation burst of more than 3.0. It is worth noting that these three keywords – “double blind,” “randomized controlled trial,” “clinical trial,” and “trial,” – almost represented the same clinical approach and had high citation bursts of more than 1.90 from 2013 to 2019.

**TABLE 6:** The top 20 high-frequency keywords.

Rank	Keywords	Count	Centrality	Rank	Keywords	Count	Centrality
1	Electroacupuncture	115	0.24	11	Therapy	18	0.11
2	Blood pressure	57	0.26	12	Injury	18	0.04
3	Myocardial ischemia	46	0.14	13	Nervous system	18	0.06
4	Heart	30	0.15	14	Rostral ventrolateral medulla	15	0.05
5	Stimulation	29	0.15	15	Disease	15	0.07
6	Rat	29	0.08	16	Activation	14	0.04
7	Management	24	0.14	17	Heart rate	14	0.15
8	Mechanism	23	0.10	18	Reperfusion	13	0.07
9	Acupuncture pretreatment	23	0.13	19	Angina	12	0.08
10	Cardiovascular disease	23	0.25	20	Prevention	12	0.09

## Discussion

### General information

Signs of prosperity have not formed, with no more than 100 publications each year from 2011 to 2021. In the past, for a long time, acupuncture was used to treat neurological diseases (including hemiplegic paralysis, facial paralysis, insomnia, and cephalalgia), orthopedic diseases (such as protrusion of the lumbar intervertebral disc, cervical spondylosis, and knee osteoarthritis), and gastrointestinal diseases (such as constipation, diarrhea, and hiccup singulation); cardiovascular diseases were not included in the spectrum of common ailments treated by acupuncture (16). Due to the characteristics of acute onset, severe condition, and high mortality of cardiovascular diseases, as a complementary and alternative therapy, the therapeutic effect of acupuncture in cardiovascular diseases has been neglected in the past. In recent years, with the development of the mechanism of acupuncture in analgesia, anti-inflammatory and nerve regulation (7–9), and effects in withdrawal syndrome (17), weight loss (18), and blood pressure regulation (19), acupuncture has become a potential therapeutic method for reducing cardiovascular disease risk factors, reducing the number of acute attacks, improving the results of electrocardiography, and alleviating anxiety and depression (20). This could explain the slow development progress of this field in the past, but the trend of steady growth augurs well for the future.

Considering the number of outputs and centrality, China and the United States are the leading countries where research on acupuncture for treating cardiopathies occurs and has maintained a certain degree of cooperation with other countries. As the birthplace of acupuncture, China accounted for 63.6% of all publications. Unfortunately, there is not much cooperation between China and the United States. Therefore, researchers and institutions from China and the United States should remove academic barriers and seek more cooperation for high-quality and in-depth studies. Network analysis of institutions’ publications can help to identify the most influential groups in this research field. The three institutions with the most publications were Beijing University of Chinese Medicine (39 publications), Chengdu University of Traditional Chinese Medicine (28 publications), and China Academy of Chinese Medical Sciences (26 publications). As shown in Figure 3, the thin connecting lines between these three nodes represent no ideal cooperation between these institutions. The lack of communication and cooperation among the most productive institutions will certainly limit development of the field. Hence, it is strongly recommended to strengthen cooperation and exchanges of research findings between institutions, reduce academic barriers, and promote the development of acupuncture for treating cardiopathies.

From the perspective of the authors, Fanrong Liang, a Chinese scholar from Chengdu University of Traditional Chinese Medicine, is the most productive, with 21 publications. Fanrong Liang’s team committed to clinical efficacy research of acupuncture for the treatment of angina pectoris. An randomized controlled trial (RCT) investigating electroacupuncture on chronic stable angina pectoris was published in *JAMA Internal Medicine* (IF = 20.768) (21). From the top ten productive authors, Cunzhi Liu, Yu Wang, Jingwen Yang, Guangxia Shi and Liqiong Wang all came from Beijing University of Chinese Medicine. This team from Beijing University of Chinese Medicine focused on clinical and mechanistic research of acupuncture-related therapies (electroacupuncture and transcutaneous electrical acupoint stimulation) in the treatment of hypertension (including essential hypertension and stage 1 hypertension), arrhythmia, stable angina, and myocardial ischemia.

*EVID-BASED COMPL ALT* was the most popular journal, with 50 publications, and ranked second in the co-cited journals, which means it plays a key role in this research field. The journal *Circulation* ranked first in the number of co-cited journals with a high impact factor (IF = 29.69). The average impact factor of the top ten journals was 2.07, and that of the top ten co-cited journals was 16.64. Based on support from high-quality references from leading journals around the world, higher quality articles are expected to be published in the future.

### Research hotspots

Keywords summarize topics, research techniques, and core content of research. Through keyword co-occurrence analysis, keyword clusters and keyword citation bursts, we can trace the development of various hotspots in a particular field. Combined with the cluster titles in Figure 7, we summarized the following three research hotspots, in which the rostral ventrolateral medulla (RVLM) and autonomic nervous system (ANS) were the most popular mechanisms of acupuncture for treating cardiopathies.

#### Blood pressure regulation and mechanism

With the deepening of research on acupuncture and neuroendocrine network theory, acupuncture gradually became a new star in the regulation strategy of blood pressure. Strictly speaking, hypertension belongs to vascular illnesses among cardiovascular diseases. However, considering that chronic hypertension often leads to hypertensive heart disease, hypertension was also included in this study as a risk factor for heart disease.

Several clinical trials have proven that acupuncture therapies [including abdominal acupuncture (22), auricular acupuncture (22, 23), electroacupuncture (24), and transcutaneous electrical acupoint stimulation (25)] have therapeutic effects on multiple types of hypertension [prehypertension and mild hypertension (26, 27), renal failure-induced hypertension (28), hypertension in stroke patients (29, 30), and hypertension in perimenopausal women (19, 31)]. However, more than 5 meta-analyses suggested that, subject to a high risk of bias, undesirable sham acupuncture control and insufficient treatment and follow-up period, there was no evidence for the sustained blood pressure lowering effect of acupuncture, which is required for the long-term management of chronic hypertension (32–37). Further RCTs are expected to involve more scientific sham acupuncture devices, more rigorous methodological settings, and longer follow-up periods. The determination of the antihypertensive effect of acupuncture will provide a positive effect on the prevention and treatment of hypertensive heart disease.

Acupuncture plays a therapeutic role through a wide variety of pathways. For example, in spontaneously hypertensive rats, inhibition of sympathetic nervous system activity can lower blood pressure. Acupuncture can increase neuronal nitric oxide synthase (nNOS) expression in the arcuate nucleus (ARC) and ventrolateral periaqueductal grey (vlPAG) to inhibit the sympathetic nervous system, thereby lowering blood pressure (38). In addition, acupuncture inhibits the sympathetic nervous system via the regulation of renal sympathetic and ss-adrenergic receptor expression (39). Other mechanisms include oxidative stress (40), the contraction of vascular smooth muscle (41), the endogenous opioid system (42) and intestinal microbiota (24). In neuroimaging studies, acupuncture stimulation was related to several brain regions (43) in which the RVLM is responsible for the regulation of blood pressure and sympathetic output (44, 45). The RVLM is correlated with the antihypertensive effect of various types of hypertension (46–48), but the reflex pathway and central neurotransmitters need to be further explored. Fan et al. (46) summarized the clinical and mechanistic studies of acupuncture in the treatment of hypertension in detail (49), and the summary and reflection on the current research indicate the direction of future research.

#### Coronary heart disease

The therapeutic effect of acupuncture on coronary heart disease, including angina pectoris, myocardial ischemia and myocardial ischemia–reperfusion injury, is another research hotspot.

In the study of acupuncture treatment for stable angina pectoris, clinical research was the majority, and systematic reviews and meta-analyses emerged steadily. Remarkable clinical evidence came from Chengdu University of Traditional Chinese Medicine in China. A total of 404 patients with chronic stable angina pectoris were treated with acupuncture, pseudoacupuncture and conventional treatment. It was found that acupuncture at the Neiguan and Tongli acupoints could significantly reduce the frequency and severity of angina pectoris attacks (21). With the emergence of high-quality clinical evidence, acupuncture was increasingly recognized as an effective adjunct therapy of anti-angina drugs, whereas studies with adequate blinding and a valid sham control group are needed.

Myocardial ischemia has been studied extensively in animal experiments; an isoproterenol-induced myocardial ischemia model and a left anterior descending coronary artery (LAD) ligation model are commonly used. In the selection of acupuncture strategies, pre-treatment and post-treatment were both used by researchers, but no study focused on the therapeutic differences between them. PC6 (Neiguan) was the most commonly used intervention acupoint. Acupuncture has a definite effect on myocardial ischemia and ischemia–reperfusion injury, but the underlying mechanism is still unclear and disputable. For chronic myocardium ischemic injury, electroacupuncture achieved cardioprotective effects through activation of the phosphoinositide 3-kinase/Akt signaling pathway (50), regulation of the gene expression of KATP and protein kinases (51), modulation of sympathetic and parasympathetic nerve remodeling (52), and regulation of gut microbiota (53). Mechanisms of acupuncture for ischemia–reperfusion injury include but are not limited to cardiac sympathetic regulation (54), pyroptosis (55), oxidative stress (56) and macrophage polarization (57). Zhang et al. (58) summarized the mechanistic studies on acupuncture for treating myocardial ischemia–reperfusion injury before 2020 and provided a theoretical and methodological basis for further clinical applications (58).

#### Regulation of heart rate and variability

Heart rate variability (HRV) refers to the fluctuations between each beat of the heart and represents the body’s flexibility and adaptability to changes in the external environment. HRV is a reflection of ANS function and is considered a breakthrough to study the physiological effects of acupuncture (59, 60). The intervention methods mainly involved electroacupuncture and auricular acupuncture (27, 61–63), and research objects included women with primary dysmenorrhea (64, 65), people at high risk of stroke (66), hypertension patients (27), and atrial fibrillation patients (67). Related mechanisms include increased basal aortic sympathetic nerve activity (CSNA), vagus nerve activity, parasympathetic vagal tone and reduced sympathetic stress (27, 62). Negative results that did not support the effect of acupuncture on HRV still existed (68, 69), and thus, clinical evidence and mechanistic studies with higher quality are expected in the future. To date, there are no outstanding scholars or institutions in this small field, and there is no representative review that discusses the development process or prospects in detail.

### Highlights and limitations

To the best of our knowledge, this is the first study summarizing the research progress on acupuncture in cardiopathies by bibliometric analysis, intuitively presenting contributors, collaboration networks, research hotspots, and development prospects through visualization. Future scholars can understand the development status and identify development prospects by reading this paper, and by learning about contributing scholars and institutions in this field, further cooperation and exchanges are expected to increase. In addition, the specific information of the 321 articles included in the analysis is provided in the Supplementary Material, and representative literature is also listed in the paper. Based on the above information, scholars can quickly identify high-quality studies for further research.

There are still some limitations in this study. First, subject to the limitations of data analysis in CiteSpace, we only collected literature from the WoS Core Collection Database; we did not include literature from Chinese or other English databases. Some original Chinese studies of systematic reviews and meta-analyses were not included in the scope, which had an impact on the results. The conclusion would be more persuasive when combined with other databases. In addition, given the purpose and length of the study, the specific therapeutic effect and mechanisms of acupuncture on related diseases cannot be discussed in detail.

### Perspectives for future studies

First, skepticism remains about the effectiveness of acupuncture on cardiopathies, limited by many problems in designing and implementing existing trials. This is a common challenge in RCTs of acupuncture, and fortunately, scholars have focused on this issue and developed guidance for designing high-quality clinical trials, covering complete methodological recommendations (70). Therefore, future clinical studies about acupuncture on cardiopathies are expected to provide high-quality evidence for clinical practice and Medicare reimbursement decisions with rigorous methodological design. In addition, RVLM and ANS will be hotspots of mechanistic research in future studies on acupuncture in cardiopathies. The RVLM is a major node in the neural network responsible for blood pressure regulation and heart rate adjustment (71, 72). The ANS network is an important part of the cardiovascular nerve regulation system, and the heart is doubly innervated by the sympathetic and vagus nerves (73). Current studies have preliminarily confirmed the relationship between acupuncture treatment and RVLM and ANS (27, 43–45, 59, 60, 62), but the specific reflex pathways, central neurotransmitters, or biomarkers need to be further explored with brain localization technology, functional imaging technology, and neuroimaging technology.

## Conclusion

Using CiteSpace for bibliometric analysis, this study can help researchers identify the research process and frontiers in acupuncture therapy on cardiopathies over the past decade, and undoubtedly lays a solid foundation for further research in this field. The three most striking frontiers are acupuncture for blood pressure regulation and hypertensive heart disease, acupuncture for coronary heart disease, and acupuncture for regulation of heart rate. It is worth noting that the RVLM and ANS have the potential to become hotspots in further studies.

## Author contributions

XoL, ZY, and FYL were responsible for the concept and design. XoL, ZY, FYL, QZ, XnL, and WQ drafted the manuscript and critically revised the manuscript. XnL, WQ, and FRL approved the manuscript. All authors made a significant contribution to the work reported and approved the submitted version.
